# Highly active electrocatalysts of iron phthalocyanine by MOFs for oxygen reduction reaction under alkaline solution†

**DOI:** 10.1039/d0ra03468a

**Published:** 2020-07-20

**Authors:** Chengcheng Wang, Bingxue Hou, Shuxian Yuan, Qi Zhang, Xumei Cui, Xintao Wang

**Affiliations:** Shen Zhen Polytechnic Shenzhen 518055 China wangchengcheng@szpt.edu.cn wangxintao@szpt.edu.cn; Aviation Engineering Institute, Civil Aviation Flight University of China GuangHan 618037 China; Panzhihua University Panzhihua 617000 China; School of Optoelectronic Technology, Chengdu University of Information Technology Chengdu 610225 China

## Abstract

Metal–nitrogen–carbon materials (Fe–N/C) have been extensively studied as one of the most excellent electrocatalysts with good catalytic activities and cheap price towards the oxygen reduction reaction (ORR). The rational design of metal–organic framework (MOF) derived carbon materials with rapid mass transport ability and good stability is a great challenge to achieve. Herein, intensive research of Fe–N/C catalysts prepared from assembling MOFs with cheap iron phthalocyanine (FePc) for the ORR is innovatively carried out. A series of Fe–N/C nano-architectures are simply synthesized by a convenient assembling method under different temperatures (800 to 1000 °C). The assembly method at high temperatures tunes the number of FeN_*x*_ active sites and intensifies the exposure of interior active sites. The highly dispersing Fe20–N/C electrocatalyst treated at 900 °C exhibits remarkable stability and excellent ORR activities with a half-wave potential of 0.866 V (*vs.* RHE) in alkaline solution, which is higher than that of commercial Pt/C (0.838 V *vs.* RHE) under the same test conditions. X-ray photoelectron spectroscopy results illustrate that incorporated MOFs interact with the active centre of FePc, tend to enhance the electron transition and to promote the kinetics of the ORR. Overall, highly dispersed Fe–N/C MOF-based materials are excellent non-precious metal electrocatalysts for energy and environmental applications.

## Abstract 2

Rational design of FePc and MOFs can result in good nanoarchitecture Fe–N/C catalysts with improved ORR activity and stability.

## Graphical abstract

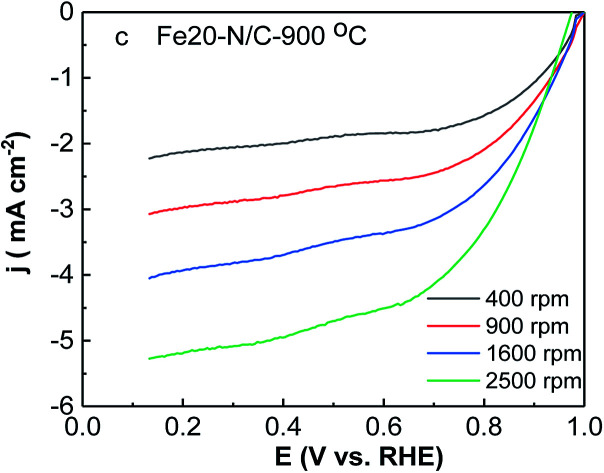

## 1 Introduction

With the increase in energy demands and environmental pollution problems, fuel cells have been emerging as one of the most promising alternatives to provide sustainable and clean energy conversion ways. However, the oxygen reduction reaction (ORR) is a four-electron or two-electron reaction and kinetically sluggish, which is almost five orders magnitude slower when using Pt electrocatalysts.^1,2^ Moreover, take fuel cells for instance, given the scarcity and expensiveness of Pt, it can account for nearly 40% of the total cell cost.^3^ Therefore, it is being paid much more attention to develop cheap, abundant as well as high active alternatives like non-precious group metal catalysts (NPMCs) including metal oxides,^4–6^ perovskite oxides,^7–11^ sulphides,^12^ carbides,^13,14^ M–N/C (M = Fe)^15–19^ in the past decade. Among NPMCs mentioned above, Fe–N/C composites are being regarded as most potential alternative electrocatalysts due to their special electronical performances and high atomic utilization efficiencies.^20–23^

Metal–organic frameworks (MOFs) are excellent candidates for metal–carbon nanocomposites due to containing a variety of metal ions and organic ligands.^24,25^ There is a strong interaction between metal and N atoms, which could benefit from inducing much more abundant N/C or M–N/C catalytic active sites. Gang Wu *et al.*^26^ firstly reported a way to synthesize active Fe–N/C catalysts that were characterized with well-uniform Fe distribution. Fe was doped into zeolitic imidazolate framework (ZIF-8). Fe–N/C electrocatalysts showed good ORR activity with half-wave potential of 0.82 V under acidic condition. Qingtao Liu *et al.*^27^ also recently reported that Fe(ii)-doped ZIF-8 was synthesized by one-step solid synthesis with no solvents, which exhibited good ORR activity under alkaline and acidic condition. Although there have been paid much attention on MOF-derived catalysts above, many MOF-derived catalysts still suffered from poor stability for ORR. Hence, it is essential to rationally design MOF-derived carbon materials with good mass transport and porous structure.

Cheap iron phthalocyanine (FePc) exhibits excellent ORR activity in M–N/C macrocycle molecules.^28^ Fe–N_*x*_ is playing a pivotal role in improving ORR activity of catalysts. However, FePc molecules are easy to aggregate and their electron conductivity does not facilitate electron transfer for ORR. Great efforts have been made to solve these problems, for instance, FePc has been supported on a wide variety of carbon materials including carbon nanotubes, graphene and so on, in order to improve its catalytic performance. Cheng *et al.*^29^ reported a simple way to incorporate FeO_*x*_ NCs by FePc functionalized graphene, FeO_*x*_/FePc, and the obtained catalyst of FeO_*x*_/FePc with 10 wt% FeO_*x*_ NCs exhibited high ORR activity with a half-wave potential of 0.888 V and current density of 37.6 A g^−1^ at 0.9 V (*vs.* RHE). Recently, Guo *et al.*^30^ also revealed that graphene-based preparation of FePc nanostructures were constructed by wet chemical method, and Fe(ii)Pc/Fe(iii)Pc/reduced graphene oxide nanocomposites exhibited good ORR activity and durability under alkaline condition. Despite extensive efforts mentioned above, synthetic methods still lack accurate control of metals, resulting in a wide variety of multispecies like metal oxides and so on.

Herein, we rationally designed a simple process that could easily control the metallic species of the MOF-based electrocatalysts with porous structure to enhance the mass transport. FePc was assembled into ZIF-8 pore structure, and was treated at high temperatures to form dispersed Fe–N_*x*_ site. With 0.20 wt% Fe doped metal, Fe20–N/C catalyst treated at 900 °C exhibited good ORR activity with half-wave potential (0.866 V *vs.* RHE) under O_2_-saturated 0.1 M KOH alkaline condition, which was higher than Pt/C-JM (0.838 V *vs.* RHE). X-ray absorption spectroscopy a results illustrate that incorporated ZIF-8 interacted with the active centre of FePc, enhanced the electron transition and promoted the kinetics of the ORR. Moreover, these highly dispersing Fe–N/C electrocatalysts fired at 900 °C exhibited excellent stability in comparison with noble Pt/C catalysts.

## 2 Experimental

### 2.1 Raw chemicals

Zinc nitrate hexahydrate, 2-methylimidazole were ordered at Sigma-Aldrich, and phthalocyanine iron (FePc) was purchased from Aldrich. Methanol, ethanol, KOH and H_2_SO_4_ were all ordered from Beijing Chemical Reagent Company, and they were all analytical grade. Nafion solution (5 wt%) was purchased from Alfa company. Ultrapure water was used. For comparison, 20 wt% Pt/C electrocatalyst (Johnson Matthey) was utilized.

### 2.2 Catalysts preparation

Typical preparation procedure of ZIF-8 can be found in the previous paper.^31^ The detailed preparation method of FePc-20/ZIF-8 can be seen below. 3.6 mmol Zn(NO_3_)_2_·6H_2_O dissolving at methanol (40 mL) was mixed with 28.71 mmol 2-mim and 20 mg FePc dissolving in 40 mL methanol solution. Then they were followed by stirring 24 h. The as-prepared powder was centrifuged and washed by methanol, which was dried at 70 °C for 24 h. For comparison, FePc-15/ZIF-8 (15 mg FePc) and FePc-25/ZIF-8 (25 mg FePc) powders were also prepared under the same experiment condition.

FePc-20/ZIF-8 powder was put inside a furnace and heated at 800, 900 and 1000 °C at 5 °C min^−1^ under N_2_ flow for 3 h. Obtained powders were treated by 0.5 M H_2_SO_4_ acid wash for 24 h at 70 °C, and they were followed by washing with ultrapure water and obtaining Fe20–N/C-800 °C, Fe20–N/C-900 °C and Fe20–N/C-1000 °C. Fe15–N/C-900 °C and Fe25–N/C-900 °C were also obtained under the same condition above. For comparison, ZIF-8 powder was put inside a furnace and heated by 800 °C at 5 °C min^−1^ under N_2_ flow for further 3 h.

### 2.3 Catalysts characterizations

The phase of prepared samples was characterized by XRD (Bruker D8 Advance) in step scan mode of 5–80° (2*θ*) for 0.02°. XPS was tested using ESCALAB 250Xi instrument from Thermo Fisher. TEM analysis was tested on Titan G2 60-300 microscope. STEM analysis was tested on Tecnai G2 F20 U-TWIN microscope. BET specific surface areas were obtained by Micromeritics TriStar II instrument.

Electrochemical performances were tested by three electrode method by Ivium work station. Inks were prepared by mixing 5 mg electrocatalysts and 1 mL ethanol–Nafion composite solution, which were then dipped on 5 mm diameter glassy carbon disc electrode. Pt wire was regarded as counter electrode, moreover, Hg/HgO was used as reference electrode.

ORR of electrodes was tested by linear scan voltammetry (LSVs) in O_2_-0.1 M KOH with scan rate at 50 mV s^−1^ due to catalysts loadings of 0.38 mg cm^−2^. Oxygen gases were purged for 30 min to make electrolyte saturated with oxygen. Linear scan voltammetry was tested at 10 mV s^−1^ under O_2_-saturated 0.1 M KOH from 400 rpm to 2500 rpm with same catalysts' loadings. Chronopotentiometry tests were done at constant potential (0.75 V) as well.

*iR* correction as well as corrected LSV curves including were done, which were shown in the previous paper. Pt/C from Johnson Matthey (Pt/C-JM) was studied for ORR under the same condition. Potentials were given *versus* RHE reference electrode (*E*_RHE_ = *E*(Hg/HgO) + *E*_Hg/HgO_ + 0.059 × pH, where *E*_Hg/HgO_ = 0.165 V *vs.* RHE at 20 °C).

Transferred electron number (*n*) was achieved by typical Koutechy–Levich (K–L) equation shown:
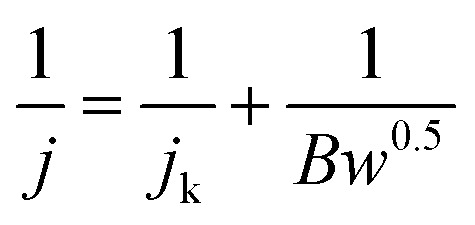
*j*_k_ and *w* were kinetic current and rotating rate. *B* was achieved from slope of K–L:*B* = 0.2*nF*(*D*_O_2__)^2/3^*ν*^−1/6^*C*_O_2__

## 3 Results and discussions

### 3.1 The effect of composition on the structural and morphology of the catalysts

**Fig. 1:**
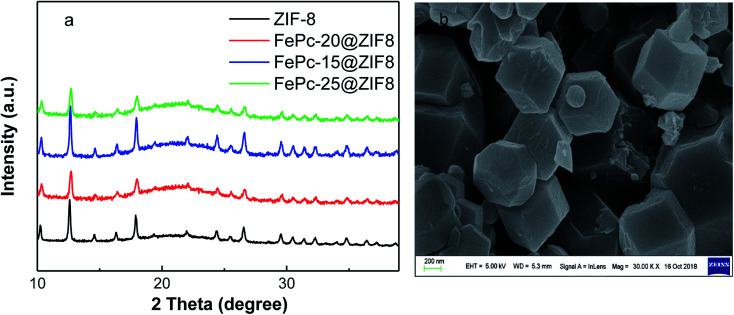
XRD of ZIF-8, FePc-20@ZIF-8, FePc-15@ZIF-8 and FePc-25@ZIF-8 (a), SEM images of ZIF-8 (b).

FePc molecules were encapsulated into cavities of ZIF-8 during assembling of Zn^2+^ and 2-methylimidazole to form FePc-*x*@ZIF-8 (*x* = 15, 20, 25 mg), respectively. FePc with the molecular size of 14.6 Å could break the ZIF-8 network with the diameter of 11.6 Å, which played a pivotal role in the construction of mesopores and edge site engineering. Fig. 1 illustrates XRD patterns of ZIF-8, FePc-*x* mg@ZIF-8 before firing (*x* = 15, 20, 25). It can be easily seen that ZIF-8, FePc-15@ZIF-8, FePc-20@ZIF-8 and FePc-25@ZIF-8 all exhibited well-defined diffraction peaks corresponding to MOF (JCPDS no. 15-0806) in XRD (Fig. 1a). The diffraction peaks didn't change with decreasing *x*, and results can indicate that the effect of FePc amount had little influence on the formation of ZIF-8, which was in accordance with previous published papers.^32,33^Fig. 1b clearly shows SEM image of ZIF-8, and it demonstrated that the good dodecahedral shape and uniform sizes of ZIF-8. The average particle size of ZIF-8 was around 300–400 nm.

**Fig. 2:**
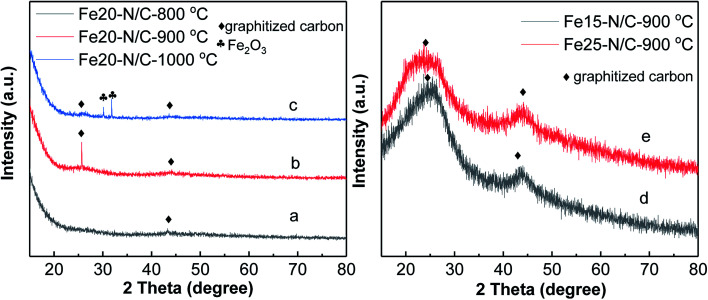
XRD patterns of Fe20–N/C samples obtained from the pyrolysis of FePc-20@ZIF-8 fired at 800 °C (a), 900 °C (b), 1000 °C (c), Fe15–N/C (d) and Fe25–N/C (e) samples obtained from pyrolysis of FePc-15@ZIF-8, FePc-25@ZIF-8 fired at 900 °C after acid treatment.

Fig. 2 shows XRD patterns for Fe20–N/C catalysts derived from direct pyrolysis for FePc-20@ZIF-8 at 800 °C, 900 °C and 1000 °C (a–c). Obvious graphitized carbon (JCPDS: 41-1487) peaks can be observed for three samples. Moreover, at 1000 °C, very minor Fe_2_O_3_ peak can be found at 31 and 32° with JCPDS number 49-1346 (Fig. 2c). That might be related to the reason that the appearance of Fe_2_O_3_ phase was related to the temperature. Moreover, XRD patterns of Fe15–N/C and Fe25–N/C catalysts fired at 900 °C were also exhibited in Fig. 2d and e. It can be seen that graphitized carbon peaks were observed as well, although the spectrums were not as smooth as those detected in Fe20–N/C catalysts fired at different temperatures. That might be related to the testing parameter difference when collecting XRD data.

### 3.2 The element valence analysis of the catalysts

X-ray absorption spectroscopy analysis was performed to study the chemical state and the coordination environment of Fe active centre. The compositions of Fe20–N/C treated catalysts derived from direct pyrolysis of FePc-20@ZIF-8 from 800 °C to 1000 °C were further investigated by XPS. The selected survey scan of the catalysts was shown in Fig. S1a,† indicating the presence of O, N and C elements. For samples treated at 800 °C, a small peak with binding energy of 500 eV might was related to the impurity substances occurred during the XPS test. Fig. S1b† shows the selected survey scan of Fe15–N/C and Fe25–N/C catalysts firing at 900 °C, which can also indicate the presence of O, N and C elements and they were the same as those observed in Fig. S1a.†

**Fig. 3:**
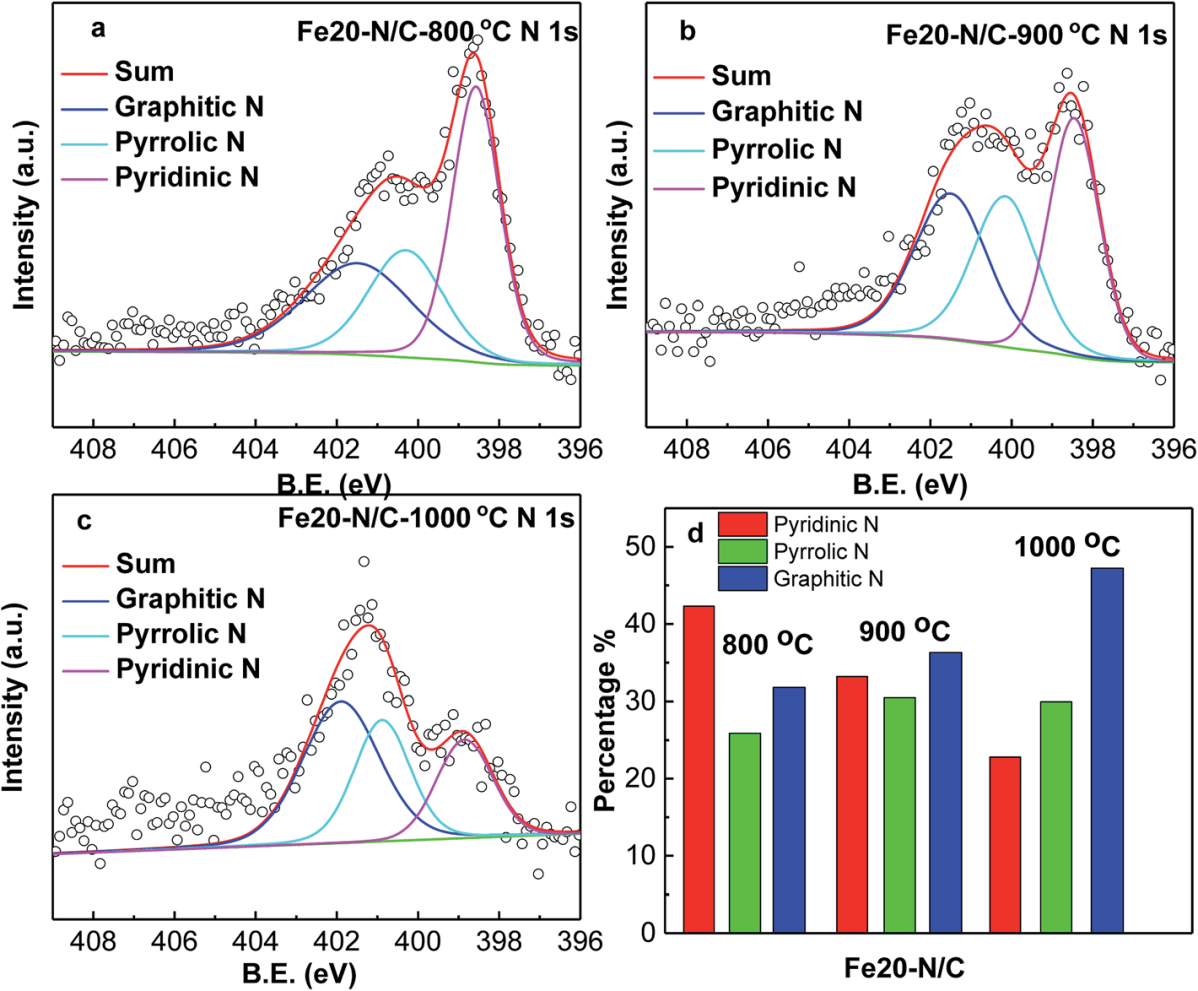
High resolution nitrogen 1s of XPS spectra of Fe20–N/C samples fired at 800 °C (a), 900 °C (b) and 1000 °C (c), normalized ratios of pyridinic, pyrrolic, graphitic N (d).

**Fig. 4:**
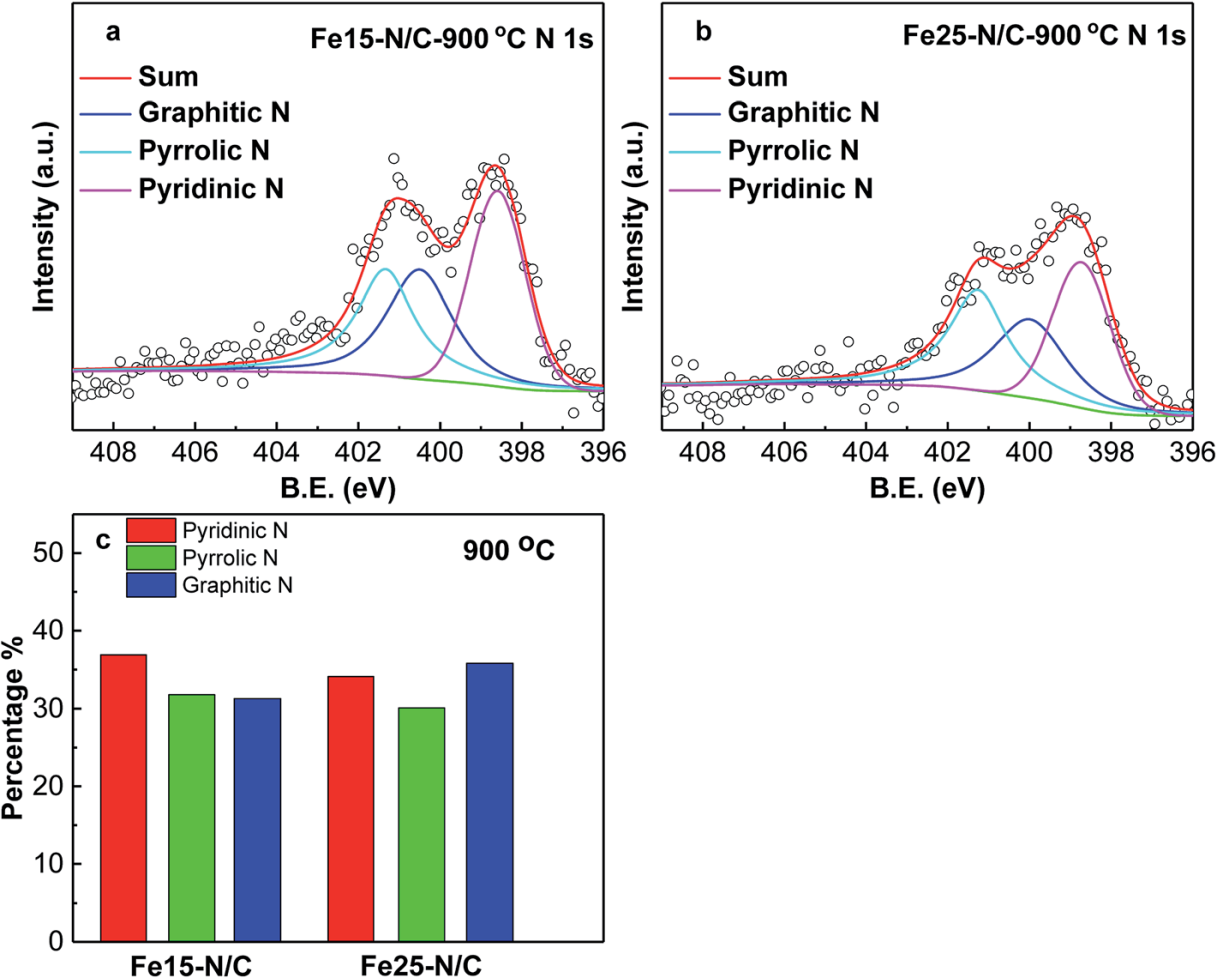
High resolution nitrogen 1s of XPS spectra of Fe15–N/C (a), Fe25–N/C (b) samples fired at 900 °C. Normalized ratios of pyridinic, pyrrolic, graphitic N (c).

**Fig. 5:**
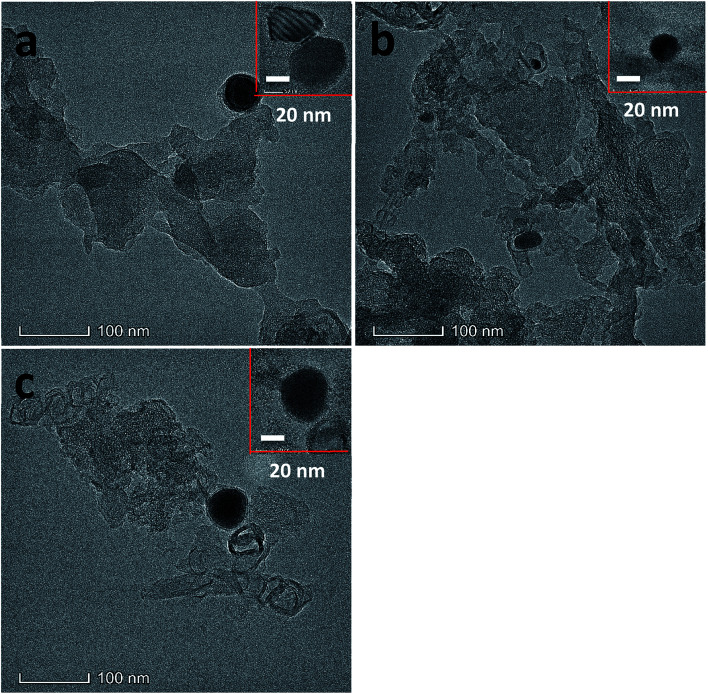
TEM image of Fe20–N/C samples fired at 800 °C (a), 900 °C (b) and 1000 °C (c).

Fig. 3 shows deconvoluted N 1s peaks for Fe20–N/C treated from 800 °C to 1000 °C. For three samples, pyridinic, pyrrolic as well as graphitic N with binding energy of 398.46–398.85 eV, 400.16–400.88 eV and 401.48–401.9 eV were clearly deconvoluted and content ratios of pyridinic, pyrrolic as well as graphitic N in all nitrogen species bonds were clearly marked for three samples in Fig. 3d. The high resolution deconvoluted N 1s peaks for Fe15–N/C and Fe25–N/C treated at 900 °C were also shown in Fig. 4. Fig. 5c clearly shows content ratios of pyridinic, pyrrolic and graphitic N in all nitrogen species bonds. The high contents of pyridinic and graphitic N was excellent due to be beneficial for generating higher positive charge density carbon atoms nearby and increasing limiting current density by benefiting from O_2_ adsorption and weakening of O=O bonds. The change from the content of pyrrolic to graphitic N with increasing from 800 °C to 1000 °C, which was similar with published reports on N-doped carbon materials.

Fig. S2† shows high resolution deconvoluted C 1s peaks for Fe20–N/C treated catalysts derived from direct pyrolysis of FePc-20@ZIF-8 from 800 °C to 1000 °C. Fig. S3† also shows deconvoluted of C 1s peaks of Fe15–N/C and Fe25–N/C treated catalysts fried at 900 °C. For three samples, C=O, C–N and C–C peaks of binding energy 289.0–289.3 eV, 285.5–285.7 eV and 284.7–284.78 eV were clearly deconvoluted and the content ratio of C=O, C–N and C–C were clearly marked for three samples in Fig. S2d.† It can be observed that the content ratio of C–N in all C species of the nanocomposites increased from 800 °C to 900 °C, and C–N decreased at 1000 °C, which could be related to cleavage of C–N bonds during pore formation.

### 3.3 The high-resolution morphology analysis of catalysts

**Fig. 6:**
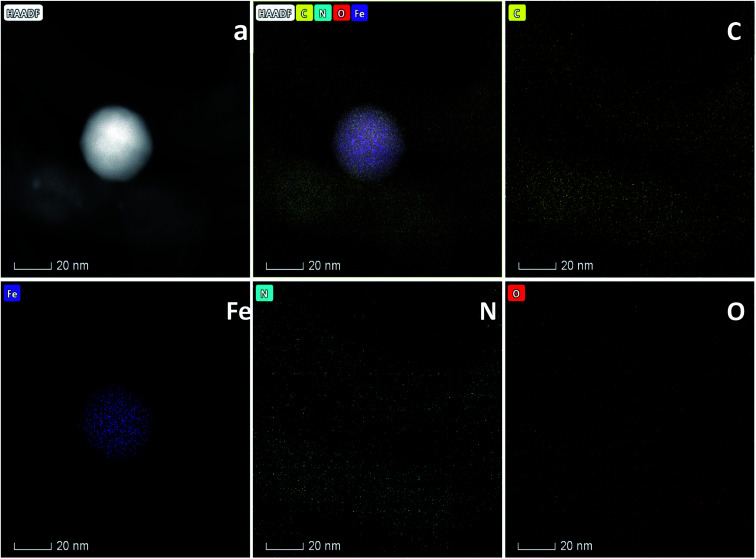
Elemental analysis of Fe20–N/C samples fired at 900 °C and corresponding element mapping files. (a) STEM image.

**Fig. 7:**
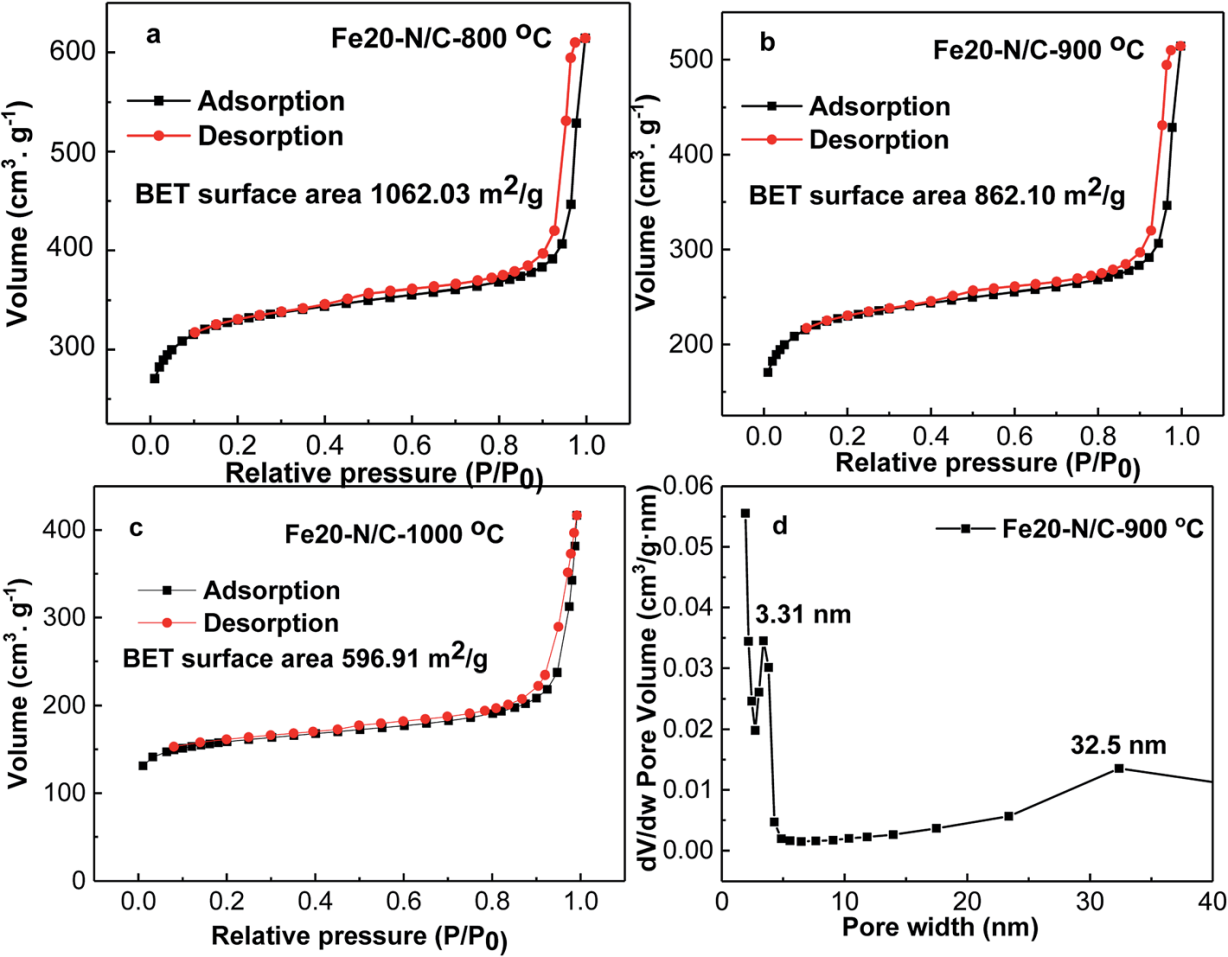
N_2_ adsorption/desorption isotherms of, Fe20–N/C samples fired at 800, 900, 1000 °C (a–c), DFT pore size distribution of selected sample (d).

The morphology of Fe20–N/C treated catalysts derived by direct pyrolysis of FePc-20@ZIF-8 from 800 °C to 1000 °C were characterized by TEM in Fig. 5. TEM analysis indicate that hexagonal particle size was around 30 nm in dimension in Fig. 5a. By increasing firing temperature to 1000 °C in the synthetic process, the shape of the particles in the final catalysts gradually changed from hexagonal to round particles. Elemental mapping analysis of selected area of Fe20–N/C catalyst (big particle as shown in Fig. 5b) fired at 900 °C was observed in Fig. 6. Iron nanoparticles generated probably due to the additional amount of FePc and some ruptures were formed in porous carbon after firing at 900 °C. FePc was chosen as the metal nitrogen precursor because it can not only create adjustable densities of metal sites but also modulated the carbon pore size. Fig. 7 showed the N_2_ adsorption/desorption isotherms of Fe20–N/C treated catalysts derived from direct pyrolysis of FePc-20@ZIF-8 from 800 °C to 1000 °C with BET of around 1000 m^2^ g^−1^. It can be shown in Fig. 7d that the mesopores (pore width > 2 nm) developed in Fe20–N/C with pore size around 3.31 nm to 32.5 nm. The construction of mesopore is mainly due to FePc bursting the ZIF-8 cage for the sake of breaking the confinement effect of micro-cavity.

### 3.4 The electrocatalytic performance of the catalysts

**Fig. 8:**
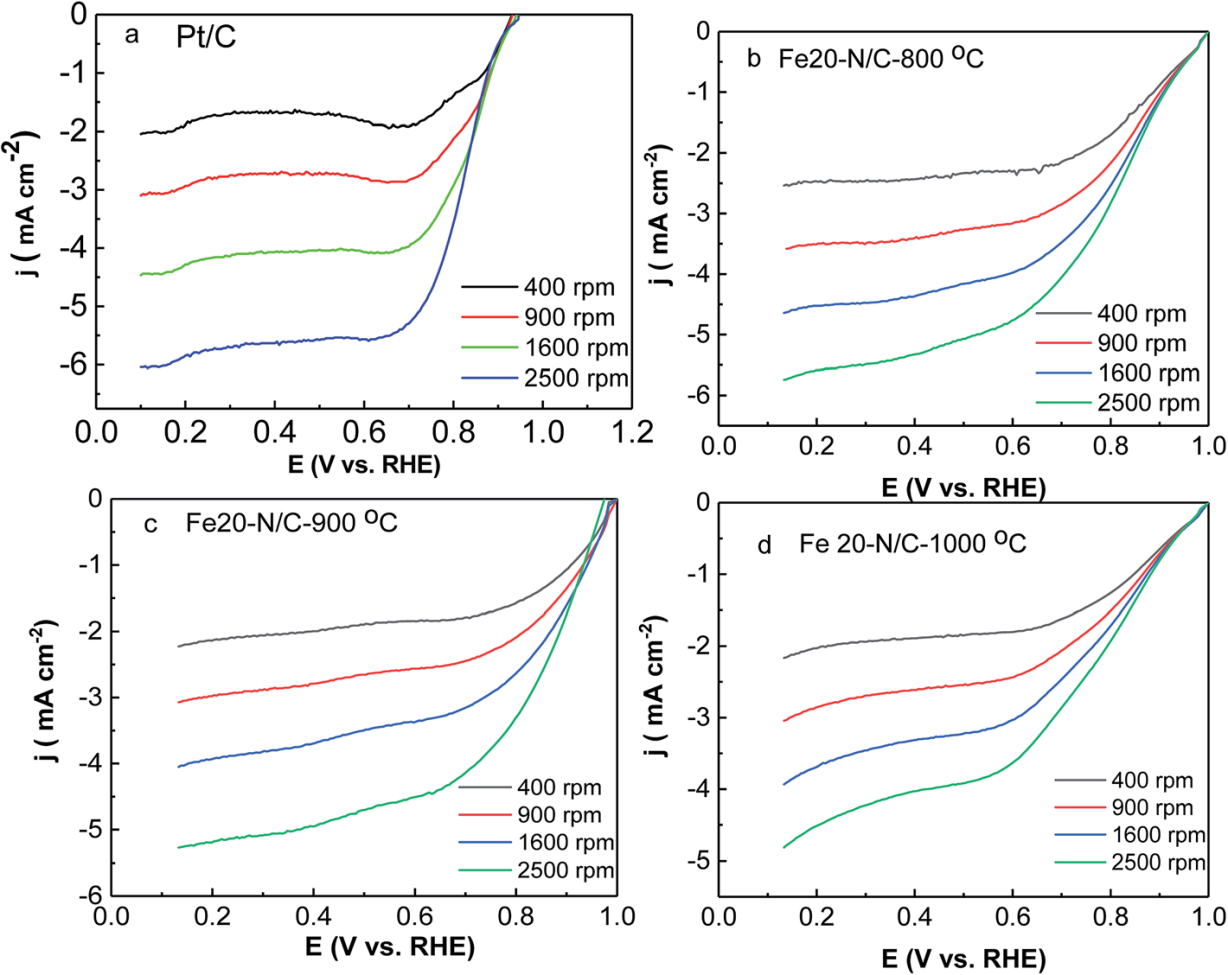
Liner sweep voltammetry (LSV) for oxygen reduction of Fe20–N/C samples fired at 800 °C (b), 900 °C (c), 1000 °C (d) for 3 h and Pt/C (a) in O_2_-0.1 M KOH at different rotation speeds with scan rate (5 mV s^−1^). The loading of catalysts was 0.38 mg cm^−2^.

ORR activity of Fe–N/C nanoarchitectures electrocatalyst was tested by rotating disk electrode measurements under O_2_-saturated 0.1 M KOH solutions. The activity of Fe20–N/C treated catalysts obtained from direct pyrolysis for FePc-20@ZIF-8 at 800 °C, 900 °C, 1000 °C and Pt/C electrodes were studied by LSV under O_2_-saturated 0.1 M KOH solutions at 10 mV s^−1^ from 400 rpm to 2500 rpm (Fig. 8). When the rotation rate was 1600 rpm, onset potential was 0.95 V, while half-wave potential of Fe20–N/C-800 °C was 0.818 V, respectively. Moreover, Fe20–N/C-900 °C exhibited much better ORR performance (onset potential 0.98 V and half-wave potential 0.866 V). And it was 48 mV more positive than Fe20–N/C-800 °C, which can indicate the obvious effect of temperature on electrochemical performance of catalysts. However, when temperature increased to 1000 °C, onset potential negatively shifted to 0.95 V, half-wave potential changed to 0.772 V (Fig. 8d). Noticeably, half-wave potential of Fe20–N/C-900 °C was 28 mV more positive than Pt/C-JM. So, Fe20–N/C-900 °C exhibited excellent ORR performance under this testing condition. Half-wave potential value (0.866 V) was relatively higher in NPMCs reported in Table 1. Chen *et al.* reported an atomically Fe–N/C and exhibited onset potential (0.95 V) and half-wave potential (0.85 V) due to catalyst loading of 0.6 mg cm^−2^.^34^

**Table 1:** ORR activities for NPMCs reported in literature^a^

Materials	Testing condition		ORR		References
	Electrolyte (0.1 M KOH)	Loading (mg cm^−2^)	*E*_onset_	*E*_half-wave_	
FePc/rGO	0.1 M	0.53	0.940	0.855	35
Co/N–C	0.1 M	∼0.25	0.834	0.75	36
Atomically dispersed Fe–N–C	0.1 M	0.6	0.95	0.85	34
Co–N/C	0.1 M	0.6	0.95	0.85	31
Fe20–N/C-900 °C	0.1 M	0.38	0.98	0.866	In this paper

a *E*_onset_ and *E*_half-wave_ is obtained based on the linear scan voltammetry results from literature.

**Fig. 9:**
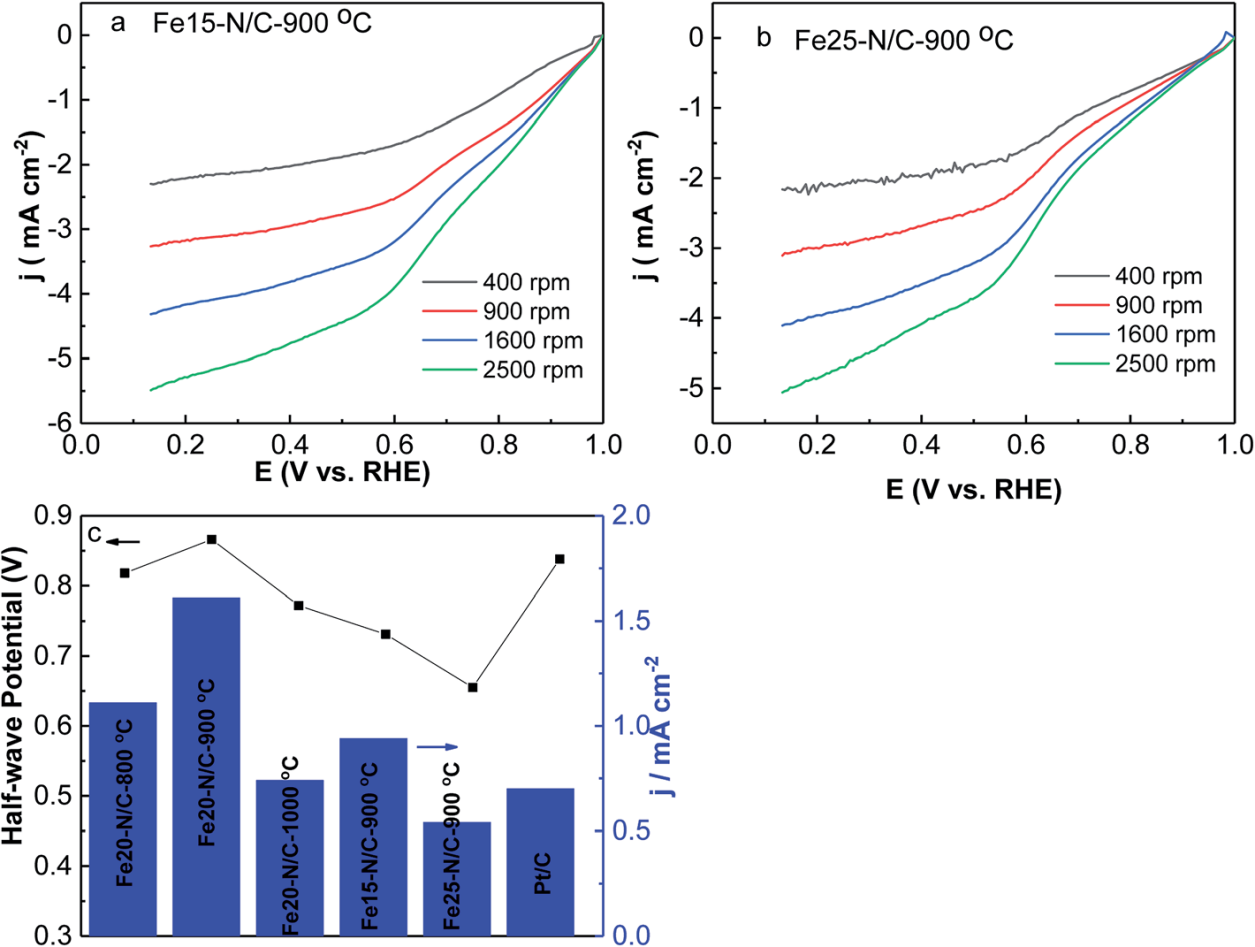
LSV for oxygen reduction of Fe15–N/C, Fe20–N/C (a), and Fe25–N/C (b) samples fired at 900 °C, Pt/C in O_2_-0.1 M KOH with different rotation speeds with scan rate (5 mV s^−1^). The loading of catalysts was 0.38 mg cm^−2^. Half-wave potential and exchange current density obtained at 0.9 V for Pt/C, Fe20–N/C samples fired at 800 °C, 900 °C, 1000 °C and Fe15–N/C, Fe20–N/C, and Fe25–N/C samples fired at 900 °C (c).

K–L curves (Fig. S4†) illustrated first order reaction kinetics towards concentrations. All samples exhibited quite low H_2_O_2_ yield and electron transfer number of 4 (Fig. 8). This suggests that 4e^−^ reduction was dominant in ORR activity of Fe20–N/C-800 °C, 900 °C and Fe20–N/C-1000 °C, with higher *n* and lower H_2_O_2_^−^%. The excellent ORR performance of Fe20–N/C-900 °C was supported by Fig. 9c. The cathodic current density of Fe20–N/C-900 °C was 1.61 mA cm^−2^, which was 0.91 mA cm^−2^ higher than 20 wt% Pt/C. ORR activity was 0.5 mA cm^−2^ and 0.87 mA cm^−2^ higher than Fe20–N/C-800 °C and Fe20–N/C-1000 °C, respectively.

The effects of different amounts of FePc on ORR activity of Fe*x*–N/C (*x* = 15 mg, 20 mg and 25 mg) treated catalysts derived from the direct pyrolysis of FePc-*x* mg @ZIF-8 at 900 °C were studied by LSV under O_2_-saturated 0.1 M KOH from 400 rpm to 2500 rpm (Fig. 9). It is worth mentioning that Fe20–N/C-900 °C showed significantly better ORR performance than Fe15–N/C and Fe25–N/C, which exhibited onset potential (0.98 V) as well as for half-wave potential (0.866 V). It can indicate the optimum amount of FePc loading was 20 mg and it could exhibit a positive effect on the electrochemical performance of catalysts.

**Fig. 10:**
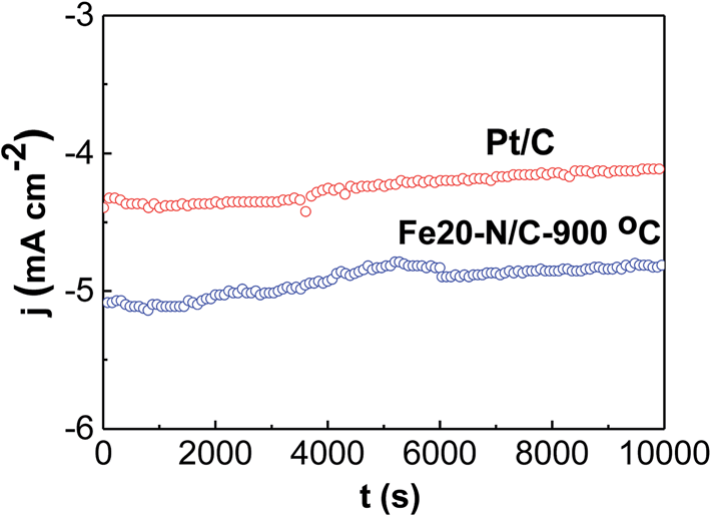
Current time chronoamperometric curves of Fe20–N/C catalyst fired at 900 °C as well as Pt/C at 1600 rpm in O_2_-0.1 M KOH at 0.75 V. The catalyst loading was 0.38 mg cm^−2^.

Stability for the Fe20–N/C treated at 900 °C and Pt/C for ORR were studied at constant potential for 0.75 V by chronopotentiometry (Fig. 10). Fe20–N/C-900 °C displayed remarkable stability for ORR, achieving potential for 0.75 V of 5.1 mA cm^−2^ during start point and achieved to 4.9 mA cm^−2^ after 10 000 s, which can indicate that current slightly decreased. The activity for Pt/C were investigated, the initial current was 4.3 mA cm^−2^, and significantly, the current to obtain potential of 0.75 V after 10 000 s polarization was 4.1 mA cm^−2^ respectively for Pt/C, respectively. Our results demonstrate that Fe20–N/C-900 °C (0.38 mg cm^−2^) exhibited more excellent performance than Pt/C. These results demonstrated remarkable performance of Fe20–N/C-900 °C electrode as nonprecious group metal catalysts with excellent ORR activity and stability.

### 3.5 Discussion

Highly dispersing Fe20–N/C electrocatalysts fired at 900 °C exhibited remarkable stability and excellent ORR activities due to the assembling effect of FePc and MOFs. There are several reasons that can account for this. One of the main reasons was probably related to the structure of FePc and ZIF-8. FePc with the molecular size of 14.6 A could break the ZIF-8 network with the diameter of 11.6 A, which played a pivotal role in the construction of mesopores and edge site engineering. As it can be shown in XRD results, the effect of FePc amount had little influence on the formation of dodecahedral ZIF-8. By rationally design of FePc molecules encapsulating into cavities of ZIF-8 could result in good nanoarchitecture Fe–N/C catalysts with improved stability.

Another reason may be due to the results that incorporated ZIF-8 interacted with the active centre of FePc, Fe–N_*x*_, could enhance the electron transition and promoted the kinetics of the ORR, and the high contents of pyridinic and graphitic N was excellent due to be beneficial for generating higher positive charge density carbon atoms nearby and increasing limiting current density by benefiting from O_2_ adsorption and weakening of O=O bonds, which was confirmed by the XPS deconvolution result.

## 4 Conclusion

The engineering for highly dispersing Fe–N_4_ sites embedded in porous carbon was reported in this study. We have demonstrated that incorporating the ultrafine Fe–N/C electrocatalyst with hexagonal nanostructures can improve ORR activity of FePc-based electrocatalysts. Fe20–N/C firing at 900 °C was demonstrated to be excellent non-precious group metal oxygen catalysts, achieving onset potential as well as half-wave potential of 0.98 V and 0.866 V, which was better than Pt/C catalysts.

## Conflicts of interest

There are no conflicts to declare.

## Supplementary Material

RA-010-D0RA03468A-s001
