# Supplementary material for: Highly active electrocatalysts of iron phthalocyanine by MOFs for oxygen reduction reaction under alkaline solution
Source: RSC Adv. 2020 Jul 20;10(45):27014–23. doi: 10.1039/d0ra03468a (PMC9055539; doi:10.1039/d0ra03468a)
Supplement: RA-010-D0RA03468A-s001 [file RA-010-D0RA03468A-s001.pdf]

Electronic Supplementary Material (ESI) for RSC Advances.
This journal is © The Royal Society of Chemistry 2020

Supporting information

Highly Active Electrocatalysts of Iron Phthalocyanine by MOFs for Oxygen Reduction Reaction under Alkaline Solution

Cheng Cheng Wang[a,1], Bingxue Hou[b,1], Shuxian Yuan[a], Qi Zhang[c], Xumei Cui[d,*],Xintao Wang[a,**]

[a]*Shen Zhen Polytechnic, Shenzhen, 518055, China*

[b]*Aviation Engineering Institute, Civil Aviation Flight University of China, GuangHan,618037, China*

[c] *Panzhihua University, Panzhihua, 617000, China*

[d]*School of Optoelectronic Technology, Chengdu University of Information Technology, Chengdu, 610225, China.*

[1]*The authors are making the same contribution to this paper.*

[*] Corresponding Author:

wangchengcheng@szpt.edu.cn (Dr. Chengcheng Wang)

wangxintao@szpt.edu.cn (Xintao Wang)

Figure S1. Full XPS spectrum of Fe20-N/C samples fired at 800 ^{o}C, 900 ^{o}C 1000 ^{o}C (a), Fe15-N/C and Fe25-N/C samples fired at 900 ^{o}C (b).

Figure S2. High resolution C1s XPS spectra of Fe20-N/C samples fired at 800 ^oC (a), 900 ^oC (b) and 1000 ^oC (c), The relative content ratio of C-N bonds in all C species (C N bonds in all C species (C-N, C-C, and C=O) of the catalysts (d).

Figure S3. High resolution C1s XPS spectra of Fe15-N/C (a) and Fe25-N/C (b) samples fired at 900 °C. The relative content ratio of C-N bonds in all C species (C N bonds in all C species (C-N, C-C, and C=O) of the catalysts (c).

Figure S4. Fe2p XPS spectra of Fe20-N/C samples fired at 800 ^{o}C , 900 ^{o}C and 1000 ^{o}C (a) , Fe15-N/C and Fe25-N/C samples fired at 900 ^{o}C (b).

Figure S5. Corresponding Koutecky-Levich (K-L) plots derived from the LSV results on catalysts of Fe20-N/C samples fired at 800 ^{o}C, 900 ^{o}C, 1000 ^{o}C for 3 h and commercial Pt/C. The loading of catalysts was 0.38 mg/cm^{2}.
